# Larynx myofibroblastic tumor, a rare case report

**DOI:** 10.1002/ccr3.6281

**Published:** 2023-08-02

**Authors:** Borja Bazan Inostroza, Jorge Prada Pendolero, Gustavo Eisenberg Plaza, Magdalena Adrados, Eduardo Raboso García‐Baquero

**Affiliations:** ^1^ Department of Otorhinolaryngology‐ Head and Neck Surgery Hospital Universitario de La Princesa Madrid Spain; ^2^ Department of Pathology Hospital Universitario de La Princesa Madrid Spain

**Keywords:** benign, larynx, myofibroblastic, pathology

## Abstract

Myofibroblastic tumors are extremely rare in the larynx, with just over 40 published cases. Despite being a benign tumor, it presents with a marked inflammatory character, local destruction, and the possibility of degeneration to malignant histological types with metastatic capacity. Anatomopathological differential diagnosis is fundamental in these cases.

## Graphical abstract

Myofibroblastic tumor is a benign tumor with a high capacity for local destruction, malignant degeneration, and the possibility of metastasis. Anatomopathological and immunohistochemical studies are essential for diagnosis.

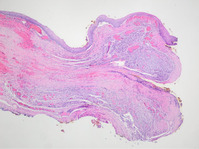

## 1 INTRODUCTION

Myofibroblastic tumors of the larynx are a very rare finding with approximately 40 cases published in the scientific literature.^1^ They are benign neoplasms with a marked inflammatory character that affect soft tissues and can become locally invasive, malignant, or metastatic. In most cases, they are asymptomatic space‐occupying lesions, which require differential diagnosis with other laryngeal neoplasms. There are several nomenclatures to define this entity, but we will use the term inflammatory myofibroblastic tumor (IMT) described by Umiker in 1954, as it is the most used name and was designated as the official nomenclature by the World Health Organization in 1994.^2^, ^3^ The term inflammatory alludes to the benign nature of this neoplasm; however, cases of advanced local invasion have been described due to the inflammatory nature of the lesion itself and degeneration to more aggressive histological types in the larynx. The most frequently described locations of this tumor are the lungs, lymph nodes, spleen, liver, mesentery, and wall of the gastrointestinal tract.^2^, ^3^

They can also affect the breast, bones, nerves, and central nervous system.^5^, ^6^

They are less frequent in head and neck areas, with described involvement of parapharyngeal spaces, oropharyngeal, nasopharyngeal, pterygopalatine fossa, infratemporal fossa, paranasal sinuses, orbit, and oral cavity.^6^, ^7^, ^8^, ^9^ In the larynx, they mainly affect the vocal cords, with cases described at the supraglottic and subglottic levels.^1^, ^10^

The pathogenesis of this entity is related to reactive processes, infections, autoimmune diseases, laryngeal trauma, and other neoplasms, with no clear etiology described.^8^, ^11^ It affects mostly children and young adults.^5^

Most cases described at the laryngeal level are asymptomatic. They may cause dysphonia, dyspnea, pharyngolaryngeal foreign body sensation, or dysphagia, among others.^12^

## 2 METHOD

**FIGURE 1:**
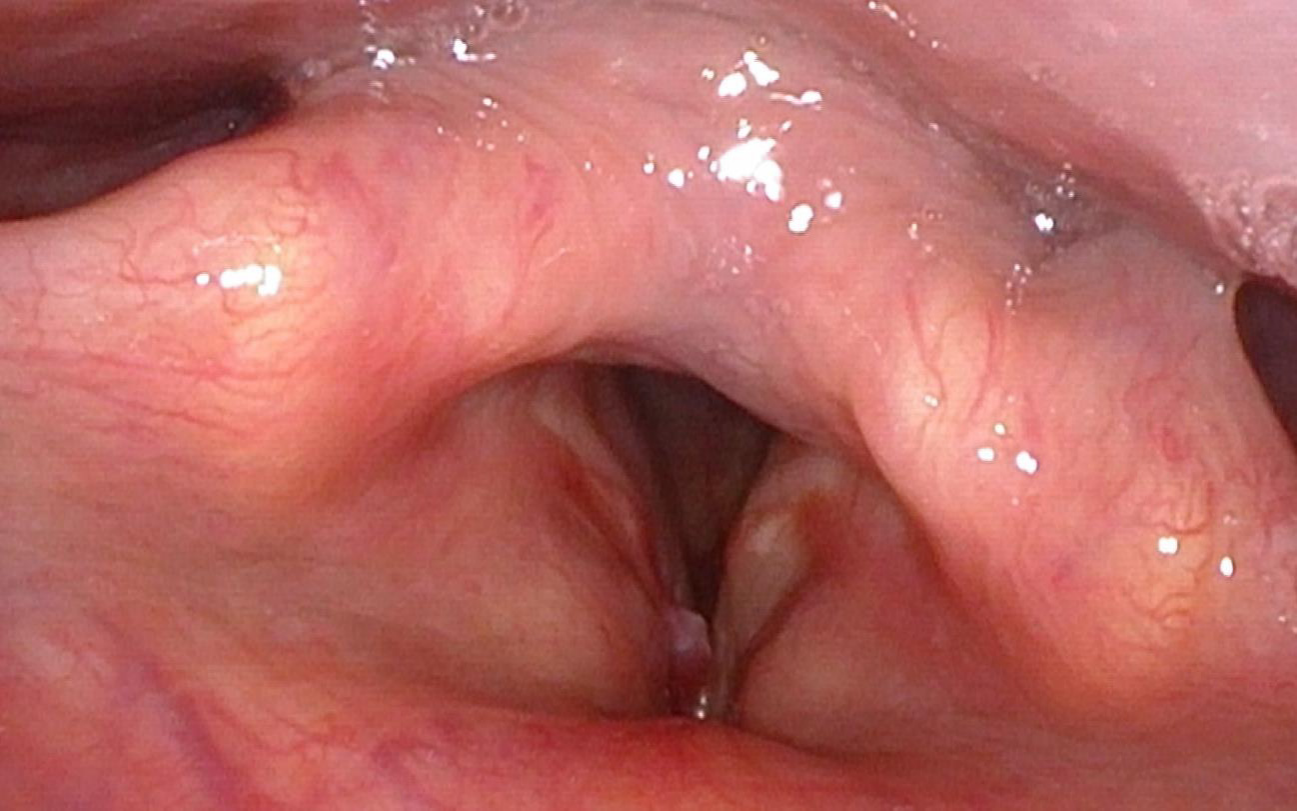
Fibrolaryngoscopic examination of myofibroblastic tumor. The lesion has the appearance of an angiomatous polyp, in the anterior third of the right vocal cord, adjacent to the anterior commissure

We present the case of a 39‐year‐old man who had been seen at another center with clinical symptoms of dysphonia that had been evolving for months. He was referred for pathological findings of an IMT of the right vocal cord after a biopsy by direct laryngoscopy. The patient had no personal records of interest or toxic habits. He was non‐smoker and non‐drinker. He did not report any consumption of illicit substances. The blood count and coagulation were normal, with the only abnormal finding in the biochemical study: being LDL cholesterol 280 mg/dL. During the examination, the patient presented symptoms of dysphonia and pharyngolaryngeal foreign body sensation along months of evolution. The fibrolaryngoscopic examination revealed a lesion with the appearance of a pedunculated angiomatous polyp <1 cm in its longest axis, in the anterior third of the right vocal fold, without involvement of the anterior commissure (Figure 1). The mobility of the right vocal cord was sparing. A NBI light scan was performed to identify superficial vascular changes of the vocal cord. A lesion with a longitudinal vascular pattern with sinusoidal and ectatic changes was identified. These findings were suggestive of benign pathology according to the classification proposed by the European Society of Laryngology. The rest of the physical examination was normal, and no cervical lymphadenopathy was observed during the cervical examination. Preoperative head and neck CT scan was performed to assess the local extent of the lesion and rule out distant metastases.

## 3 RESULTS

**FIGURE 2:**
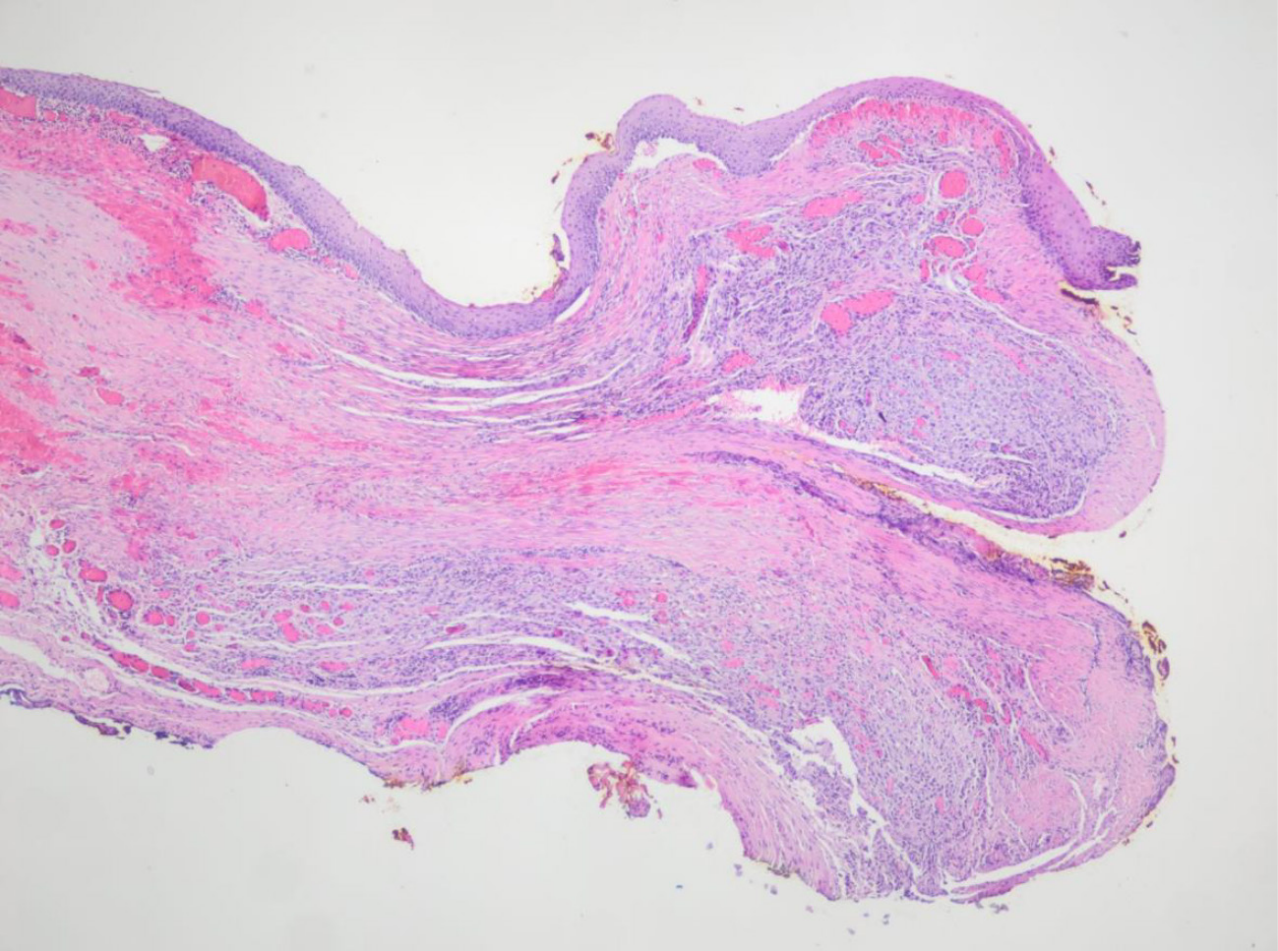
Histologically, the lesion shows increased focal cellularity at the expense of a predominantly spindle‐shaped cell population with rounded nuclei, slight hyperchromatism, and arranged in a stroma with marked vascularization

**FIGURE 3:**
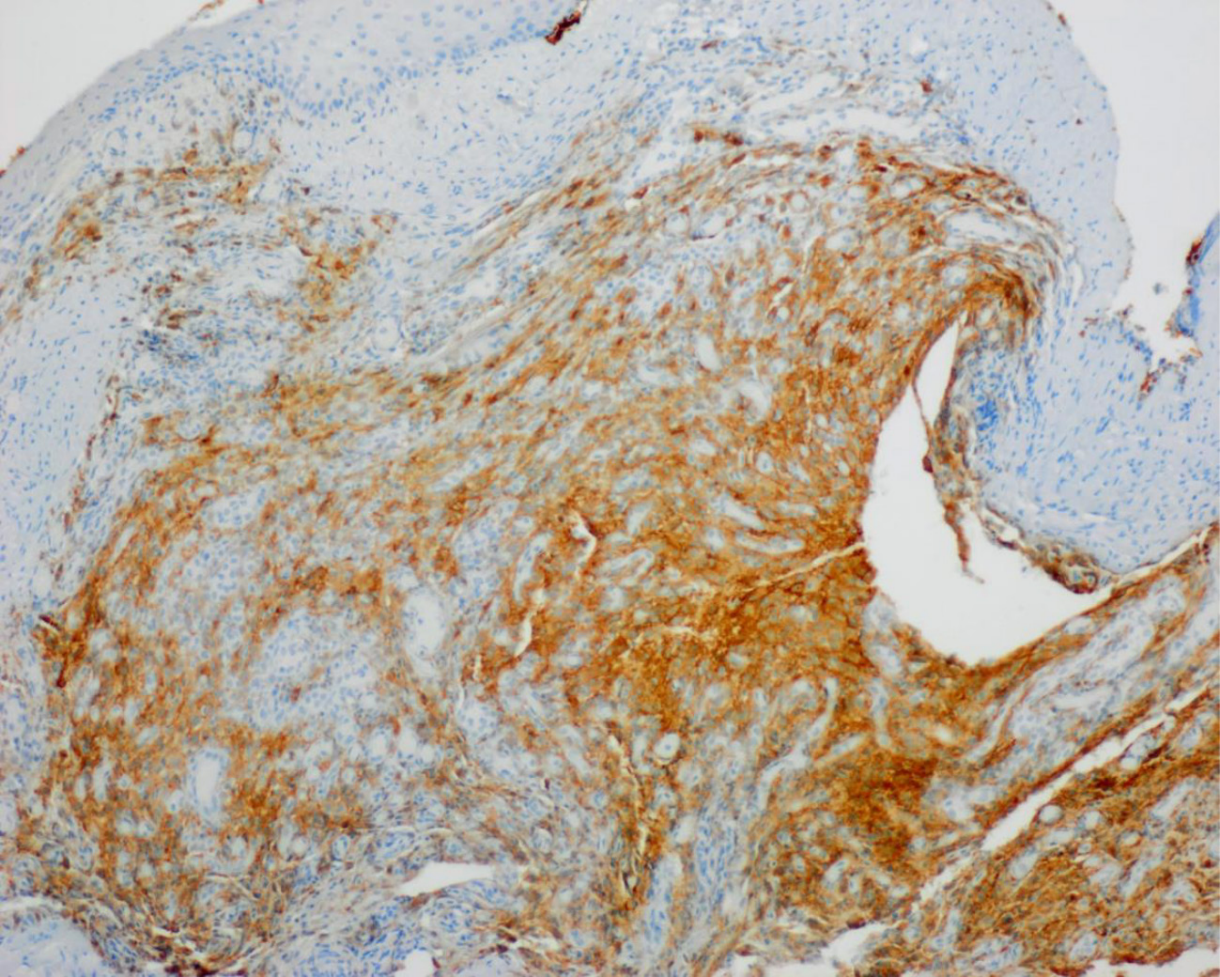
Immunohistochemistry showed focal mesenchymal proliferation with cytoplasmic positivity for ALK‐1 and negativity for smooth muscle actin

**FIGURE 4:**
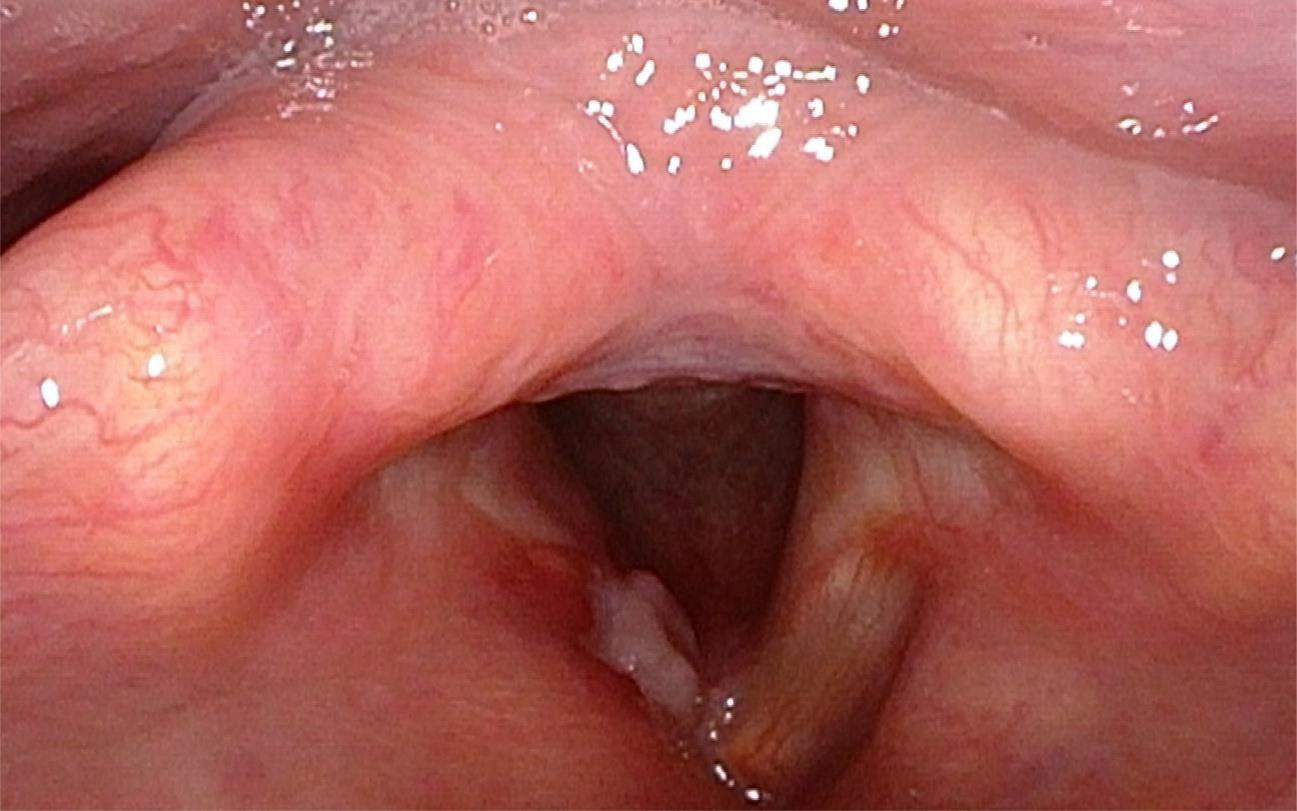
Fibrolaryngoscopic examination 6 months after surgery, with an area of nodular fibrotic appearance suspicious for recurrence in the area of surgical resection

Surgical resection was scheduled by the Otorhinolaryngology Department. Partial cordectomy was performed with CO2 laser, including complete resection of the lesion with margins up to the muscular plane of the vocal cord, sparing the anterior commissure. The anatomopathological result of the surgical specimen was focal mesenchymal proliferation with ALK expression compatible with IMT (Figures 2 and 3). The case was presented to the Head and Neck Tumor Committee of the hospital, where it was decided to follow it up by the Otorhinolaryngology Department. During the following months, a portion of surgical sequelae was observed in the area of partial cordectomy of the right vocal cord with marked fibrosis and granulation, which acquired a nodular appearance over the next months. After 6 months of follow‐up, it was decided to perform microsurgery on the larynx to take a biopsy to rule out recurrence (Figure 4). Preoperative head and neck CT scan was performed to assess tumor recurrence and rule out distant metastases. Laryngeal microsurgery was scheduled, and biopsies were obtained from the nodular fibrotic area with suspected recurrence. The pathological findings of the biopsies showed respiratory mucosa, granulation tissue, and chronic inflammatory changes of a non‐specific nature. After 8 months of follow‐up, there was no evidence of recurrence. Given the risk of recurrence and metastasis of this pathology, the Committee decided to continue close follow‐up for at least 12 months, in accordance with most of the series published in literature.

## 4 DISCUSSION

Head and neck cancer comprises malignant lesions affecting the oral cavity, pharynx, larynx, salivary glands, as well as the nasal cavity and paranasal sinuses. Worldwide, it ranks seventh among malignant neoplasms. In USA, there are more than 54,000 new cases per year with an incidence of around 15/100,000 inhabitants and 12,000 deaths attributable to this disease.^13^ According to data from the Spanish Society of Otorhinolaryngology and Head and Neck Surgery (SEORL‐CCC), about 10,000 new cases are detected each year in Spain,^14^ mainly in men between 45 and 65 years of age. The most common histology is epidermoid carcinoma, which is related to alcohol and tobacco consumption. Laryngeal tumors with histology other than squamous cell carcinoma are rare with an incidence of <1%. Most of these are mesenchymal tumors.^10^ IMT is an extremely rare subtype in the larynx but more frequent in other areas of the head and neck. Clinically, it mimics other more common laryngeal neoplasms, requiring a differential diagnosis.

Clinically, they behave as asymptomatic masses or may cause symptoms of a space‐occupying lesion, causing dysphonia, dyspnea, dysphagia, or pharyngeal‐laryngeal foreign body sensation. Locally, the inflammatory and expansive behavior of the lesion can cause great clinical variability, mimicking other neoplastic processes. Primary involvement at the laryngeal level with systemic symptoms including constitutional syndrome, fever, anemia, or thrombocytopenia has been described,^15^, ^16^ which reflects the variability and morbidity associated with this pathology. The definitive diagnosis is eminently anatomopathological.

Histologically, they are composed of lymphocytes, plasma cells, histiocytes, fibroblasts, and myofibroblasts in variable proportion, forming four basic histological patterns: predominant lymphoplasmacytic infiltrate, predominant lymphohistiocytic infiltrate, myofibroblastic infiltrate, and lymphocytic infiltrate with collagen deposition.^4^

Immunohistochemistry is characteristic of a soft tissue tumor with ALK cytoplasmic positivity in most cases, without being a specific finding for this type of tumor. Variable cytoplasmic positivity for vimentin, smooth muscle actin, muscle‐specific actin and desmin, and negativity for myoglobin, myogenin, and S100 protein are characteristic.^3^, ^17^

Although not being a specific finding, it is also characteristic to observe genetic rearrangements on chromosome 2p23 with activation of the ALK receptor tyrosine kinase gene, leading to overexpression and activation of the ALK protein. It occurs in approximately 50% of cases and appears to have an age‐dependent distribution, with this finding being more common in adults under 40 years of age. This overexpression also appears to have a morphological distribution within the tumor itself, with activation occurring in the myofibroblastic component without expression of this genetic alteration in the inflammatory cells of the tumor itself. In any case, if there is no genetic alteration in ALK, genetic rearrangements have been observed in the HMGIC gene (also known as HMGA2) on chromosome 12, t(2, 17), (p23, q23), tropomyosin 4 (TPM 4), TPM 3, t(p25, p23), cysteinyl tRNA synthetase, and Ran‐binding protein. In addition, TP53 positivity has been demonstrated in some cases, which could have prognostic influence, being more common in recurrent cases, with transformation to malignant histological types or production of metastases.^2^, ^3^, ^8^, ^12^, ^18^

Despite being very infrequent with its lesions in the larynx, IMT is a pathology that has to be considered, requiring similar procedures as laryngeal neoplasms, where biopsies need to be taken to establish a clearer anatomopathological diagnosis for defining a treatment plan. The first‐line treatment is surgery, either with cold instruments or resection with laser surgery. Radical excision with free edges is the critical prognostic factor. Radiochemotherapy is reserved for cases with positive margins or a formal contraindication to surgery. A close clinical follow‐up of this pathology is essential, with most series requiring it for at least 12 months, given the variable risk of recurrence and distant metastases.^8^, ^12^, ^15^, ^16^, ^19^, ^20^

Given the marked inflammatory component of the lesion, a second‐line corticosteroid therapy is widely validated in cases with contraindications for surgery or tumor recurrence, and it is also used for head and neck IMT in locations other than the larynx.^1^

Genetic alterations are opening new lines of research: series of treatment with ALK inhibitor crizotinib have been published in cases of ALK‐positive ITM, as a second‐line therapy for those cases of contraindication to surgery, partial resection, or positive surgical margins.^12^, ^21^

## 5 CONCLUSION

IMT is an infrequent pathology in the larynx, included in the differential diagnosis of benign laryngeal pathology. Despite this, it is essential to consider the locally expansive and inflammatory nature of this lesion, the risk of transformation to malignant histological types, and the possibility of distant metastases. Anatomopathological and immunohistochemical studies are fundamental in the differential and definitive diagnosis. The first‐line treatment is surgical resection, completed with free margins, and close postoperative follow‐up given the possibility of local and distant recurrence.

## AUTHOR CONTRIBUTIONS

Bazan Inostroza B.: involved in writing and data collection, involved in the clinical and in the surgical procedure and follow‐up of the clinical case. Prada Jorge, Eisenberg Gustavo, and Raboso Eduardo: involved in supervision and review, involved in the clinical and in the surgical procedure and follow‐up of the clinical case. Adrados Magdalena: involved with the histology images and histological analysis of the surgical specimen.

## CONFLICT OF INTEREST

There are no conflicts of interest.

## ETHICAL APPROVAL

The authors of the following article declare that there is no conflict of interest. No funding was required. The project has been approved by the ethics committee of the Hospital Universitario de La Princesa (Madrid, Spain). The patient has signed the informed consent for the study to be carried out.

## CONSENT

Written informed consent was obtained from the patient to publish this report in accordance with the journal's patient consent policy. Written informed consent was provided by the patient for publication of this case and the images.

## Data Availability

The data are available from the corresponding author upon reasonable request.
